# Utility of Reactive Species Generation in Plasma Medicine for Neuronal Development

**DOI:** 10.3390/biomedicines8090348

**Published:** 2020-09-12

**Authors:** Sarmistha Mitra, Neha Kaushik, Il Soo Moon, Eun Ha Choi, Nagendra Kumar Kaushik

**Affiliations:** 1 Plasma Bioscience Research Center/Applied Plasma Medicine Center, Department of Electrical and Biological Physics, Kwangwoon University, Seoul 01897, Korea; sarmisthacu@gmail.com; 2 Department of Anatomy, Dongguk University College of Medicine, Gyeongju 38066, Korea; moonis@dongguk.ac.kr; 3 Department of Biotechnology, University of Suwon, Hwaseong 18323, Korea; neha.bioplasma@gmail.com

**Keywords:** reactive species, neuronal growth, neuronal stem cells, neurodegeneration, gas plasma

## Abstract

Reactive oxygen species (ROS) are critical signaling molecules for neuronal physiology that stimulate growth and development and play vital roles in several pathways when in a balanced state, but they cause neurodegeneration when unbalanced. As ROS levels above a certain threshold cause the activation of the autophagy system, moderate levels of ROS can be used as treatment strategies. Currently, such treatments are used together with low-level laser or photodynamic therapies, photo-bio modulation, or infrared treatments, in different chronic diseases but not in the treatment of neurodegeneration. Recently, non-thermal plasma has been successfully used in biomedical applications and treatments, and beneficial effects such as differentiation, cell growth, and proliferation, stimulation of ROS based pathways have been observed. Besides the activation of a wide range of biological signaling pathways by generating ROS, plasma application can be an effective treatment in neuronal regeneration, as well as in neuronal diseases. In this review, we summarize the generation and role of ROS in neurons and provide critical insights into their potential benefits on neurons. We also discuss the underlying mechanisms of ROS on neuronal development. Regarding clinical applications, we focus on ROS-based neuronal growth and regeneration strategies and in the usage of non-thermal plasma in neuronal and CNS injury treatments.

## 1. Introduction

Reactive oxygen species (ROS) are group of molecules which are generated from oxygen and are very reactive in nature. The properties and activity of different reactive species as signaling molecules are most widely studied signaling molecules and evident to be responsible for both positive and negative impacts on both human physiology and disease pathology, as well as in treatment of a variety of health conditions [1,2]. Because of the double-edged sword property of ROS [3], maintaining its balance and regulation is crucial [4]. ROS is found to be an essential molecule for maintaining the normal physiology of the brain by stimulating many receptors and metabolic functions [5,6]. ROS can influence multiple aspects of neural differentiation and function, including the survival and plasticity of neurons [7], the proliferation of neural precursors, as well as their differentiation into specific neuronal cell types [8,9]. On the other hand, disruption of the ROS balance and quantity of excessive amount of ROS in a specific region of the brain may cause defects in hippocampal plasticity and learning paradigms [10].The brain is the most energy-demanding organ, requiring 20% of the body’s energy [11], and mitochondrial oxidative phosphorylation is a significant source of ROS [12]. ROS and the accumulation of ROS-related damage are also associated with aging [13], oxidized lipids [14], and DNA damage [15]. However, recent studies have provided ample evidence of ROS-regulating neuronal development and function, including the establishment of neuronal polarity, growth cone pathfinding [16], and the regulation of connectivity and synaptic transmission [17].

In recent times, non-thermal plasma (NTP) has gained popularity as a great source of reactive species. NTP is an ionized gas condition that consists of a considerable quantity of reactive oxygen (ROS), hydrogen (RHS), and nitrogen species (RNS) [18,19]. With the development of physical plasma technologies, NTP has been widely investigated in cancer treatment [20], decontamination [21], dental treatment [22], wound healing [23], and other health areas, founding a new research field called plasma medicine. Recent studies with NTP have shown that it enhances proliferation of cells, cell migration, tube forming in endothelial cells, and wound healing in vitro, which is related to plasma-generated ROS and the stimulation of several growth factors, e.g., the vascular endothelial growth factor (VEGF) [24] and fibroblast growth factor-2 (FGF-2) [25]. Since limited levels of ROS promote cell proliferation, survival, migration, invasion, and angiogenesis, but may also induce autophagy, NTP-associated ROS could activate autophagy as well. As a result, NTP may be beneficial in the treatment of neurodegenerative (NDs) diseases, but it may also be detrimental. A common pathology of diverging NDs, such as Parkinson’s (PD), Alzheimer’s (AD), and Huntington’s (HD) diseases, is the presence of misfolded proteins and the accumulation of denatured proteins inside or outside of cells [26,27]. Accumulated proteins usually generate protein aggregates and eventually cause neurodegeneration. Emerging evidence suggests that NDs develop due to defects in autophagy regulation [28,29]. Therefore, activation of autophagy has been proposed as a potential mechanism to clear abnormal protein aggregations [30,31], and thus as an effective way to cope with NDs. Applying a limited amount of NTP to initiate autophagy, without causing any cell or tissue damage, could constitute a potential treatment for NDs.

Although the role of NTP in neuronal growth and development has not been deeply studied, a few works have reported that cold plasma could improve differentiation of neuronal stem cells and increase neuronal regeneration following trauma [32,33,34]. Another modulator of neuronal outgrowth and regeneration following injury can be found in glial cell microenvironment. Glial cells contain different non-neuronal cells, including astrocytes, that promote neuronal health and viability by sustaining homeostasis and by assuring support and protection for neurons. In a healthy central nervous system (CNS), astrocytes maintain neuronal health by secreting proteins and neurotrophic factors [35], and plasma application has been shown to have a significant effect on astrocyte growth. However, the studies performed on neuronal growth, development, and disease treatment using NTP have not been enough to determine the underlying mechanism of action. In addition, there are a considerable number of factors that should be considered for further research studies. The objective of this review is to highlight the role of reactive species in neuron growth and development and to focus on the underlying mechanism by which NTP acts on neuronal growth, differentiation, and regeneration to promote post-injury healing, together with functional regeneration.

## 2. Role of ROS Generated in the Neuronal Environment

The term ROS defines a group of reactive free radicals that originate from molecular oxygen (O_2_). ROS, such as the superoxide anion (O_2_•^−^), hydrogen peroxide (H_2_O_2_), singlet oxygen (^1^O_2_), and the hydroxyl radical (HO•), consist of radical and non-radical oxygen species generated by a limited reduction of oxygen [36]. These are species of oxygen that can exist independently with one or more unpaired electrons. ROS are highly reactive molecules that undergo several reduction reactions in normal cells [37]. To maintain the normal balance of ROS in cells, several defense mechanisms have been developed that include both enzymatic and non-enzymatic machineries. The enzymatic defense system includes glutathione peroxidase (GPX), superoxide dismutase (SOD), and catalase (CAT), whereas non-enzymatic antioxidants include glutathione (GSH), uric acid, melatonin, vitamins C and E, polyphenols, and other molecules. In the glutathione redox cycle, GPX utilizes GSH to reduce organic peroxides and H_2_O_2_, while the glutathione reductase reduces the oxidized form of GSH with concomitant oxidation of nicotinamide adenine dinucleotide phosphate [38]. In addition, SODs are enzymes that require metal cofactors for the conversion of O_2_•^−^ into O_2_ and H_2_O_2_ [39], and CAT is a heme-containing peroxisomal enzyme important in the decomposition of intracellular H_2_O_2_ [40]. CAT catalytically decomposes H_2_O_2_ into water and oxygen (*α* phase), or peroxidatively, by oxidizing alcohols, formate, or nitrate (*β* phase) [41]. ROS generation is in subtle balance with these antioxidative defense mechanisms. If this balance is disrupted, the ROS accumulation could become deleterious, causing different disease states. ROS directly activate oxidative stress responsive mechanisms.

In the brain tissue, microglia and astrocytes produce ROS and regulate synaptic and non-synaptic communication between neurons and glia. Although ROS are widely related to a number of ND pathologies, reported studies also suggest that ROS play an essential role in several physiological processes [42]. Endogenously, ROS can be produced in cellular organelles. Mitochondria and the NOX system are major contributors to cellular ROS production. Mitochondrial ROS have two sources: the mitochondrial respiratory or electron transport chain (ETC) and the mitochondrial outer membrane flavoprotein, also known as monoamine oxidase (MAO). Mitochondrial ETC is a powerful supplier of ROS during increased Ca^2+^ signaling. It has been reported that elevating Ca^2+^ and Na^+^ is sufficient to produce free radicals from isolated rat mitochondria [43]. Again, ROS are inevitable by-products of cellular respiration, during which an electron that escapes from the ETC binds oxygen to form O_2_•^−^ [44]. Superoxide anion generation occurs mainly at two points of the ETC, at Complex I (NADH dehydrogenase) and Complex III (ubiquinone-cytochrome c reductase). Thus, ROS formation is excessive due to metabolic demand and excitotoxicity.

Monoamine oxidases (MAO) are also potential sources of ROS in the brain. MAO, in the mitochondrial outer membrane, induce oxidative stress by producing hydrogen peroxide by oxidation of monoamine substrates [45,46].

ROS generation also occurs by the action of several enzymatic systems, such as lipoxygenases, xanthine oxidases, cyclooxygenases, monooxygenases, nitric oxide synthases (NOS), and NADPH oxidases (NOX). Among them, NOX has been described as a critical enzyme that utilizes molecular oxygen as substrate and regulates the production of ROS. Different research groups have reported seven NOX paralogs comprising NOX1–5 and dual oxidase 1/2 (Duox1/2) [47]. Noteworthy, NOX1 and NOX2 are overexpressed in microglia [48].

Glutamate is a well-known excitatory neurotransmitter engaged in neural function. Excessive buildup of intercellular glutamate leads to increased concentrations of ROS and RNS in neuronal cells [49]. Activation of the NMDA receptor by glutamate application to cultured forebrain neurons stimulates a localized ROS formation. It is known that glutamate reduces intracellular pH in a Ca^2+^-dependent manner [50], while NOS also plays a role in superoxide generation. In addition, stimulating NMDA receptor results in the production of superoxide [51].

**Figure 1:**
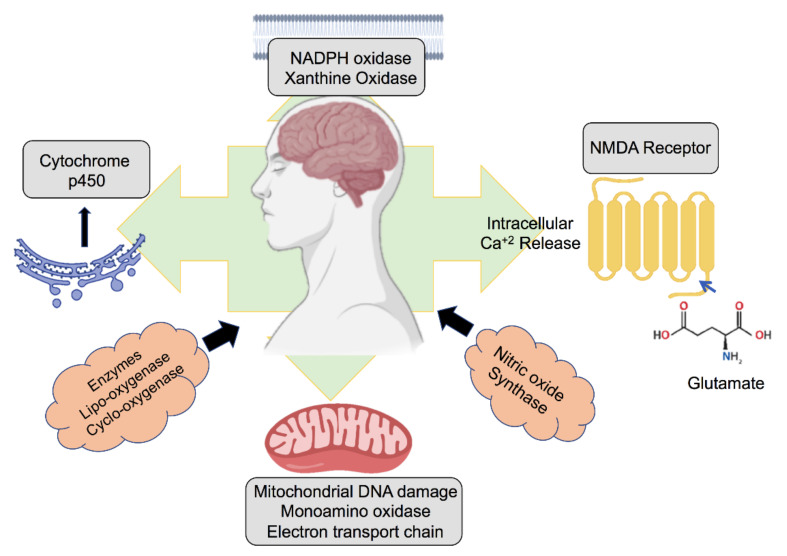
Different sources of reactive oxygen species (ROS) in the brain and neurons.

Other cytosolic sources of ROS are cytochrome *p*-450 enzymes in the endoplasmic reticulum, which generate these compounds during fatty acid oxidation. With the degradation of cellular material, ROS are moved to lysosomes via autophagic or endocytic pathways. Besides, hydrogen peroxide can freely diffuse into the lysosome from the cytoplasm. In the lysosome, low pH and high iron concentrations build a supreme environment for the formation of ROS from Fenton reactions; thus, if there is an accumulation of oxidants, lysosomes must face oxidative stress [52]. The physiological sources of ROS in the brain are shown in Figure 1.

The generation of oxidative stress resulting from an excessive production of ROS is closely related to age. As age increases, in the brain, the usual antioxidant defense machinery is reduced, leading to an increase in the susceptibility of the brain to the destructive effects of oxidative molecules [53]. It is assumed that mitochondrial DNA (mtDNA) damage is caused primarily by free radicals of mitochondrial origin. Several studies have reported elevated levels of 8-hydroxy-2-deoxyguanosine (8-OHdG) in mtDNA in the aged brain, which is a biomarker of oxidative DNA damage [54]. Besides, there is an increased accumulation of mtDNA mutations in the aging brain; thus, mitochondrial energy production-related genes become less active, and dysfunctional mitochondria are observed [55]. In addition, age per se is a risk factor that makes mitochondria more vulnerable to oxidative stress and gives rise to dysfunctional mitochondria. Eventually, these changes create a vicious cycle between mitochondrial dysfunction and oxidative damage [55]. Moreover, the “mitochondrial theory of aging” by Harman suggests that mitochondria play a vital role in aging; it indicates that aging results from accumulated damage caused by mitochondrial ROS in both cells and tissues [56].

## 3. ROS and Neurogenerative Disease Pathology

ROS have long been studied and have been established as damaging agents from the perspective of the nervous system, aging, and degeneration [57]. Oxidative stress is caused by an imbalanced production of ROS and RNS and antioxidants in cells and tissues. ROS and RNS are overproduced for several reasons, such as aging and disease, and they eventually cause cell damage by inducing chemical modifications on lipids, proteins, and nucleic acids. This type of oxidative modification may be a triggering event, ultimately leading to neuronal injury. Moreover, oxidative stress is a significant cause of neuronal disorders. Thus, excessive production of ROS/RNS has been considered a mechanism for the neurodegeneration associated with other problems of neurons, such as hypoxia and hypoglycemia [58], as well as with the neurodegeneration seen in AD [59], PD [60], and amyotrophic lateral sclerosis (ALS) [61]. In depressive disorders, oxidative stress also plays a crucial role in disease pathology. All the neurodegenerative disorders (NDs) possess similar alterations, such as abnormally aggregated protein deposition and oxidative damage caused by mitochondrial dysfunction [62]. ROS-induced oxidative stress plays a key role in the pathogenesis of AD, as it is a critical factor in A*β* peptide accumulation [63]. Moreover, oxidative damage, resulting in lipid peroxidation and nitration, reactive carbonyl species formation, and nucleic acid oxidation, is observed at increased ratios in neurons of patients with AD [64]. Cytochrome oxidase, the pyruvate dehydrogenase complex, and the α-ketoglutarate dehydrogenase complex show decreased activity as a result of oxidative damage in AD [65].

In the development of PD, ROS play a preeminent role. Excessive ROS accumulation is crucial not only in the pathology of the PD-related gene *PINK1*, but also in the physiology of PINK1/Parkin-related mitophagy [66]. PD also shows a general increase in end-product markers of oxidative and nitrosative stress, reflecting excessive damage to biomolecules. ALS shows similar hallmarks of protein accumulation and oxidative damage. Mitochondrial oxidative damage has also been demonstrated in patients affected by ALS [67]. Mutations in the ALS-related genes *TDP-43*, *FUS/TLS*, and *p62* also increase mitochondrial ROS and oxidative stress [68,69]. Apart from these neurodegenerative disorders, ROS also plays a major role in depression [70].

## 4. ROS in Neuronal Growth, Differentiation, and Synaptic Plasticity

From the discussion above, it is clear that ROS and subsequent oxidative stress are responsible for neurodegeneration, which has also been established in previous studies [71,72]. However, ROS have also been considered as regulators and modulators of signaling pathways and gene expression, many of which are known to cause neuronal growth, differentiation, and synaptic plasticity. There are redox-dependent mechanisms that promote neuronal differentiation. While the major ROS in cells are O_2_•^−^, •OH, peroxynitrite (ONOO^−^), and H_2_O_2_, recent studies have suggested that high, but sublethal, levels of O_2_•^−^ and H_2_O_2_ can control intracellular signaling pathways in neuronal cells by acting on gene expression, cellular growth, and differentiation [73,74]. As described in Section 2, NOX enzymes are a regulated source of ROS in neurons [75]. ROS production during normal neuronal development does not alter the probability of a cell of becoming a neuron, but it affects neuronal maturation in terms of morphology, physiology, and biochemistry. The possible mechanism behind this role is that ROS reversibly oxidize enzymes such as protein tyrosine phosphatases. ROS can influence tyrosine phosphorylation and subsequent signaling, controlling protein stabilization [76] during early neurogenesis and the outgrowth stage [8]. Studies also suggest that ROS, in particular H_2_O_2_, are essential for activity-induced synaptic terminal growth and sufficient to drive this process [77]. It has been found that nerve cells activate a process of self-renewal when ROS are present in the brain via the PI3K/Akt pathway [78], and it is suspected that this process may induce synapse growth. ROS can also maintain cytoskeletal changes by direct redox modification of structural cytoskeletal proteins and by indirect modification of the proteins or signaling pathways controlling cytoskeletal dynamics. All major cytoskeletal elements and cytoskeleton-associated proteins are subject to direct redox alterations [79], mainly actin, tubulin, and neurofilament fractions. Redox modification of cytoskeletal proteins likely affects other signaling pathways [80], directly or indirectly; for example, the cellular redox state guides neuronal growth cone responses to extracellular cues [81].

Similarly, stem cell differentiation is significantly controlled by the action of the redox state in various signaling pathways. Recent evidence suggests that mesenchymal stem cells (MSCs) can be used to replace injured neurons and support endogenous neuronal cell repair or survival by releasing neurotrophic factors [82,83]. Other studies also suggest that ROS-mediated neurogenesis is based on JNK signaling activation [84]. Wnt5a promotes neurogenic differentiation in human adipose derived stem cells (ADSCs), binding to Fz3 and Fz5, and signaling through the Wnt5a-JNK pathway [85]. Different members of the Wnt signaling pathway also play important roles in ROS-mediated neuronal cell differentiation, including Wnt-3a and Wnt-7a. Additionally, the Wnt/*β*-catenin pathway is activated in response to ROS [86]. Finally, Wnt can stimulate the expression of *neuroD*, *Brn3a*, and *neurogenin 1* (*Ngn1*),which are sensory neuron markers, via activation of Tlx3 [87].

Synaptic plasticity is the ability of synapses to regulate their strength, connectivity, and structure based on previously experienced activity [88]. Current evidence suggests that synaptic plasticity is regulated by both direct and indirect modes of ROS action [89]. ROS interfere with increased neuronal activity by altering the myelin basic protein. They can also induce synaptic long-term potentiation (LTP), which guides activity-dependent synaptic plasticity and memory consolidation [90]. One of the most widely studied varieties of synaptic plasticity in hippocampal LTP [91], which is a persistent increase in synaptic efficacy elicited by brief, high-frequency stimulation. This requires nitric oxide synthase activity, providing compelling evidence that nitric oxide (NO), which is an ROS, can act as an intercellular messenger during LTP. Synaptic plasticity can also be regulated by the N-methyl-D-aspartate (NMDA)-mediated pathway, where ROS play an important role [92]. In this process, NMDA receptor (NMDAR) activation causes insertion of AMPA receptors into the postsynaptic membrane. As a result, the ERK mitogen-activated protein kinase signaling cascade is activated, and it phosphorylates the cAMP-responsive element binding protein, a transcription factor that can intervene in the transcription of multiple “synapse-associated genes” necessary for memory consolidation. NMDAR activation promotes O_2_•^−^ production by NOX, which is vital for the activation of the NMDAR-mediated ERK pathway, for the full expression of NMDAR-mediated LTP, and to trigger hippocampal-dependent memory tasks [93]. Furthermore, ROS control canonical synaptic plasticity mechanisms by direct oxidative modification, by inhibiting phosphatases PP1, PP2, PTEN, and calcineurin, causing elevated kinase signaling, including those involving ERK and PKC [94].

**Figure 2:**
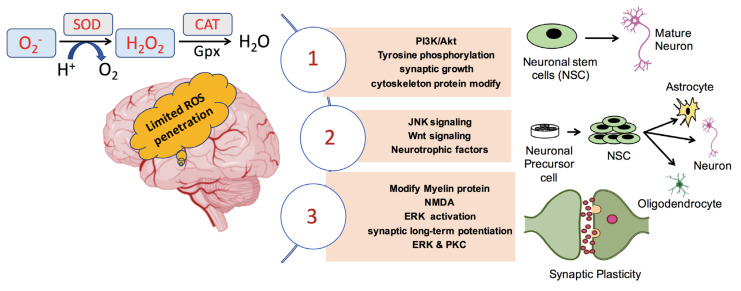
ROS mediated pathways that influence neuronal maturation and differentiation as well as synaptic plasticity in the brain.

A common pathology between diverging NDs such as AD, PD, and HD, and type II diabetes is the accumulation of denatured proteins inside or outside the cell. As previously mentioned, accumulated proteins usually form aggregates. Emerging evidence suggests that neurodegenerative diseases develop due to defects in autophagy regulation [95]. Therefore, autophagy activation has been proposed as a possible means of clearing abnormal protein aggregates [30]; consequently, as an effective way to cope with NDs. Many medicines have been designed to trigger autophagy for treating neurological disorders [96]. Physiological ROS induce autophagy [97] to maintain cellular homeostasis in different types of cells, whereas dysregulation of redox signaling can disrupt autophagic activity [98,99]. Many mechanisms and pathways are involved in ROS-induced autophagy, e.g., JNK/AP-1 pathway initiation by oxidative stress is thought to reinforce autophagy, and many genes encoding autophagy proteins are transcriptional targets of AP-1 [65]. Additionally, activation of autophagy via the oxidative stress-induced JNK/AP-1 mechanism can control synaptic terminal size and strength. Figure 2 summarizes the pathways related to neuronal growth, differentiation, and synaptic plasticity mediated by ROS. It would be of great importance if future studies were focused on the mechanisms underlying controlled autophagy initiated by ROS, aiming to assist in neuronal disease treatments.

## 5. ROS-Mediated Therapies for Neuronal Injuries

Recently, physical therapeutics whose mechanisms of action are related to ROS generation, such as different types of radiation and laser, photodynamic, non-thermal plasma, and infrared light treatments, have being used against several diseases, mainly for cancer treatment. However, a strict control of the therapeutic regimen should be followed when treating patients, given that any of these treatments can reach extreme levels of toxicity and can cause permanent damage if not carefully controlled. Such negative effects have been observed in a number of studies regarding neuronal treatment and growth induction light therapy or photo biomodulation (PBM) [100,101].

Low-level laser therapy (LLLT), also known as laser light therapy, is a method that generates shallow levels of ROS in cells, which are beneficial [102]. It is a non-thermal method of low-intensity light application. In recent studies, LLLT has been applied to neurons to treat neurotoxic [103], peripheral and central nerve, and spinal cord injuries [104], and to increase axonal growth and nerve regeneration [105]. Studies have also reported that LLLT stimulates the release of ROS, which eventually activates the NF-kB factor and releases NO in normal murine cortical neurons, resulting in a stimulation of beneficial effects, such as neuroprotection and growth [106].

Multi-watt near-infrared light therapy (NILT) is another therapeutic approach whose effects on neuronal injuries and growth have been studied [107]. Similarly, studies have focused in the mechanism of action for generating ROS and NO at beneficial levels only, those that activate the NF-kB signaling pathway and that lead to increased synaptogenesis, neurogenesis, and generation of inflammatory mediators and growth factors [108]. For NILT, the wavelength that is generally used oscillates between 800 and 1100 nm. It is evident that infrared light can penetrate the brain and has a beneficial effect on traumatic brain injury [109]. In addition, this therapy has been proven to be effective in patients suffering from anxiety or depression [110].

It has recently been shown that the human gut microbiota plays a vital role in the physiology and neurochemistry of the CNS [111]. However, the mechanism underlying the effects of microbiota on CNS disorders is yet to be discovered. Nevertheless, a recent hypothesis states that, by the influence of gut microbiota, the gut–brain axis generates physiological levels of ROS. Such controlled levels of ROS stimulate the activation of the antioxidant defense system, reducing the possibility of cell injury [112].

ROS-mediated therapies for neuronal regeneration or disease treatment may situate patients in susceptible situations because there is a thin line between having beneficial and harmful effects. Therefore, the dose of treatment and the level of ROS generation must be carefully controlled and maintained to an optimum level. Ideally, future research should be focused on the application of these therapies to patients to determine specific doses to be applied to keep them safe from possible side effects.

## 6. Application of NTP in Biomedicine

**Figure 3:**
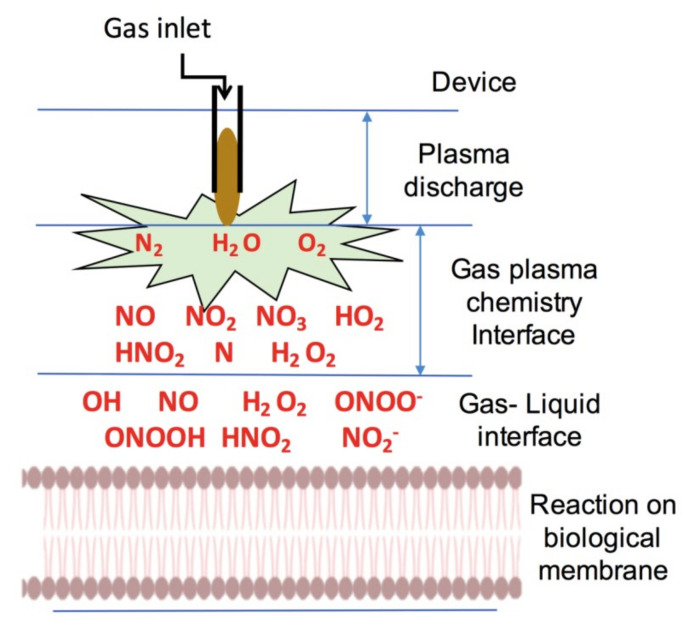
Different reactive species generated by gas plasma in air and in gas–liquid interfaces.

Non-thermal plasma, the fourth state of matter, is becoming a useful tool with an increasing number of biomedical applications; currently, it has been applied to many disease treatments such as hair loss and cancers [113]. One of the underlying mechanisms of action of NTP over biological systems relies on the fact that it is an excellent source of reactive species [114]. In liquid–air surfaces, such as those around the cell, NTP interacts with the liquid phase of the biological membrane and produces a large variety of reactive species, as demonstrated in Figure 3.

Generally, the efficiency achieved by indirect NTP application via treatment media or water depends on the generation of reactive species in liquid. When a liquid and plasma react, hydrogen peroxide, hydroxyl radicals, hydrogen radicals, singlet oxygen, nitrogen oxide, and some other species are formed [115]. The amount, nature, and types of reactive species generated by plasma mainly depend on the type of gas device used to generate the plasma [18]. In addition, the generation of reactive species can be controlled by modifying gas flow rate, plasma treatment time, plasma discharge distance, and other parameters. Beside reactive species, plasma also generates free electrons, UV radiation, and excited ions in the treated surface [116].

For biomedical applications, plasma jet devices and dielectric-barrier discharge (DBD) sources are the most commonly used devices [117,118,119]. Some of the different types of plasma devices and gases used for biomedical applications are listed in Table 1.

There is evidence that NTP enhances cell proliferation [132] and controls cell migration [133], tube formation in endothelial cells [134], and wound healing in vitro [135,136]; all these effects have been associated with plasma-generated ROS and the stimulation of growth-related factors, such as the vascular endothelial growth factor (VEGF) and the fibroblast growth factor-2 (FGF-2) [137,138]. NTP has also been used as a reliable tool for surface decontamination and sterilization of medical devices because it can inactivate microorganisms [21,116,139]. Recently, NTP has also been used in dentistry, and its beneficial effects have also been related to the generation of ROS. NTP can be used to reduce the difficulties of various dental complications, such as the elimination of caries, root canal sterilization, and bleaching. The application of this type of plasma can be done by both direct and indirect methods [140]. Several studies on the emerging field of plasma medicine have established that NTP is an emerging therapeutic agent for cancer treatment [97,141,142]. Moreover, because of the safe and effective action of plasma, research related to plasma medicine is gaining more attention. However, future research should focus on the controlled generation of different reactive species to minimize any possibility of harmful effects when treating human pathologies.

## 7. Present Scenario of Plasma Medicine Applied to Neuronal Growth

Previous studies have shown that low levels of ROS promote cell proliferation, survival, migration, invasion, and angiogenesis; therefore, NTP-associated ROS/RNS could promote neuronal growth and migration. It is also known that NDs and traumatic CNS damage are currently difficult to treat. However, neural stem cells (NSCs) can improve their treatment, and several studies have been investigating NSC proliferation induced by NTP [143].

Recent evidence suggests that NTP regulates diverse cellular processes; it can also regulate neural differentiation. However, the exact mechanisms behind the physicochemical signaling process elicited by ROS/RNS on biological systems remains elusive. Among all the plasma produced reactive species, NO plays a significant role in the CNS. NO is an essential signaling molecule required for many biological processes and plays a dual role in the physiological system, especially in the case of neurotoxicity and neuroprotection [144,145,146]. NO, at a physiologically minimal amount, can provide neuroprotection by regulating diverse signaling pathways, such as the PI3K/Akt [147] and the NO/cGMP/PKG pathways [148]. Additionally, NO is also a strong cerebral vasodilator agent [149] that can enhance cerebral blood flow (CBF) supply during ischemic brain injury or hypoxia [150].

**Figure 4:**
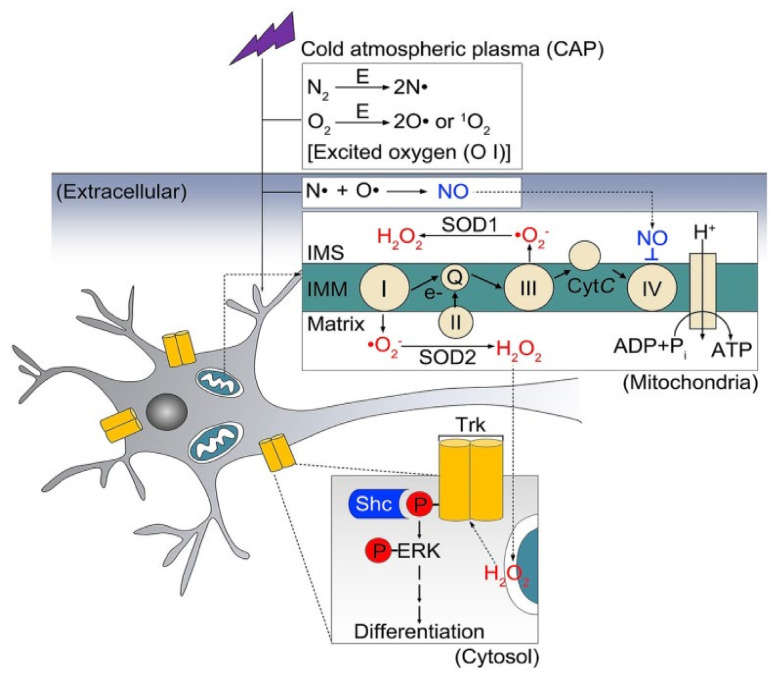
CAP-based mechanism for neuron stimulation differentiation by Trk/Ras/ERK signaling pathway [154]. IMS, intermembrane space; IMM, inner mitochondrial membrane.

Neural differentiation by plasma can have some advantages. First, the differentiation process with NBP treatment is faster than without any treatment. Second, plasma application increases differentiation efficiency noticeably by upregulating specific genes. Finally, NTP treatment can differentiate a large percentage of cells with or without other chemical inducers [151]. Interestingly, in a recent study, it was found that there are physicochemical and biological connections between the non-thermal plasma cascade and the Trk/Ras/ERK signaling pathway; what is known about the underlying mechanism is summarized in Figure 4 [152]. Besides, it was seen that the nerve growth factor exerted its effects mainly by interacting with the specific receptor TrkA [153]. Therefore, the stimulation by NTP resulted in neural differentiation. The authors considered that mitochondrial O_2_ and cytosolic H_2_O_2_ must have acted cooperatively because the experimental cytosolic increase in H_2_O_2_ by itself was not sufficient to initiate differentiation. Moreover, the mechanisms of phosphorylation of the TrkA receptor at specific sites remain unknown. Excited atomic oxygen generated in plasma eventually form reactive oxygen nitrogen species (RONS) and interact in the extracellular liquid phase with reactive atoms, generating NO. Large amounts of (O_2_•^−^) in the cell’s mitochondria exposed to plasma treatment showed that reversible inhibition of mitochondrial Complex IV is increased by extracellular NO [152].

A recent study also found that treatment by nanosecond-pulsed dielectric barrier discharge (nspDBD) plasma showed significant outcomes on astrocyte regrowth or neurite regeneration. The observed enhancement in neurite outgrowth as a result of low-intensity plasma stimulation in non-contact cocultures was probably because of soluble factors produced between neurons and astrocytes [155]. One possible mechanism of NTP enhancing neural outgrowth would be similar to that noticed in the stress preconditioning mechanism. It is evident that in the hypoxia/ischemia field that exposure to mild “mini-insults” causes injury tolerance makes neurons more resilient to damage in the future [156]. It is believed that ROS present in a low dose of NTP induce transient oxidative stress conditions, protecting cells against stronger stresses that may present later. This cytoprotective effect of non-thermal plasma has already been reported for other types of cells [157].

Au-Xiong et al., showed that NTP treatment can significantly elevate the proliferation and differentiation rates in C17.2 murine NSC lines [158]. Moreover, almost 75% of NSCs differentiated into neuronal cell lines after exposure to NTP; this percentage is higher than that achieved by specific growth factors. Differentiated neurons showed high *β*-tubulin III protein expression levels; this protein is considered a neuron marker [159]. Studies made on the usage of NTP in neuronal treatments are summarized in Table 2.

## 8. Future Perspectives

Low levels of ROS can be helpful for neuronal growth and disease management, and a number of therapies can generate a controlled amount of ROS. Photo biomodulation or PBM therapy has already been applied to several disease conditions as a successful treatment strategy [163] and has also been studied for neuronal treatments. This phenomenon needs to be further studied for optimization and for introducing it as a new strategy for neuronal regeneration. PBM and various light therapies such as infrared and laser therapies stimulate stem cell proliferation and differentiation. The underlying mechanism can be related to ROS production because the stem cell niche is hypoxic, and, when stem cells encounter ROS, their differentiation program is activated [164]. This knowledge could be applied in neuronal stem cell treatments to increase differentiation.

**Figure 5:**
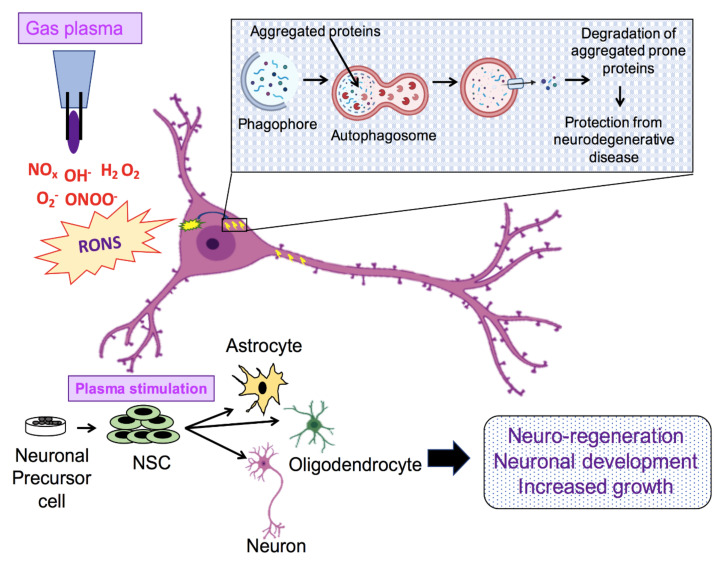
Possible mechanism of action of non-thermal plasma over neuronal differentiation and neurodegenerative diseases.

In biomedical applications and treatments, NTP has an immense potential. In various fields, NTP has already proved to be a great treatment strategy. Although in the field of neuronal growth and in the treatment of neurology-related diseases NTP has not been explored enough to find a specific mechanism of action and to describe its related effects, it can be a promising new strategy. It is already well established that NTP can generate a wide range of ROS and RNS, which is the main reason for influencing biological systems and samples. We discuss above the significance of ROS in different aspects of neural development. Thus, it can be assumed that NTP can act in the same way to initiate signaling pathways to promote neural development and neuroprotection. In addition, some studies have already reported that NTP can significantly stimulate NSC differentiation. Ideally, future studies should focus on finding the possible mechanisms by which NTP acts and how plasma can be potentially applied in growth and neural regeneration and to heal the neuronal injury or trauma post-injury (Figure 5). NTP has a very significant role in activating autophagy, which has been reported in a number of studies on different disease treatment strategies [97]. In the case of NDs, autophagy activation could play a significant role in the clearance of accumulated proteins. Therefore, NTP could be used to temporarily trigger autophagy to accelerate the clearance system. Activation of autophagy by NTP efficiently removes intracellular denatured proteins and promotes neuronal growth and development (Figure 5).

From the discussion, it is clear that plasma treatments could become successful therapeutic strategies to protect neurons from degenerative cascades. However, plasma treatments still face several challenges in the field of neuroregeneration. Recent studies have shown that plasma at low doses can enhance antioxidant activity, stimulate immune cells such as macrophages to clear plaque, and stimulate stem cells. Therefore, future plasma medicine research could focus in the study of neurodegenerative disease-targeted approaches by using non-thermal plasmas for stimulating immune and stem cells, enhancing the antioxidant capacity of cells, and improving cell–cell communication. Considering the potential of these ROS-based therapies in the neuron, future studies should be performed using in vivo models and should involve clinical studies on new therapeutics, balancing the positive and negative effects of NTP.

## Figures and Tables

**Table 1:** Different types of NTP devices used in biomedical applications.

Year	Name of Device	Gas Used	Biomedical Application	Reference
2019	KINpen Jet	Argon	Bone Cancer	[120]
2019	MiniJet-R	Argon	Bone Cancer	[120]
2019	Plasma Jet	Argon	Skin Cancer	[121]
2017	DBD	Nitrogen	Cervical cancer	[122]
2018	Micro Plasma	Helium	Breast Cancer	[123]
2018	Plasma Jet	Helium	Breast Cancer	[124]
2018	Micro Plasma	Helium	Brain Cancer	[123]
2020	Plasma jet	Helium	Prostate	[125]
2019	DBD	Helium and air	Wound healing	[126]
2018	DBD	Helium	Wound healing	[127]
2009	Plasma Jet	Helium, Nitrogen, Oxygen	Dentistry	[128]
2020	DBD	Helium	Dentistry	[129]
2012	Microsecond pulse plasma jet	Helium and Oxygen	Disinfection	[130]
2019	Surface micro-discharge plasma	Air	Sanitation	[131]

**Table 2:** Non-thermal plasma application for neuronal treatments.

Year	Plasma Device	Cell Line	Mechanism	Activity	Reference
2017	Plasma Jet	SH-SY5Y	Reducing cell apoptosis	Neuroprotection	[33]
2013	Micro-plasma jet	Neural stem cells	NO species induce gene expression	Cell Differentiation	[32]
2019	Nanosecond-pulsed dielectric barrier discharge	Cortical neurons	Stress preconditioning mechanism	Neurite re-growth	[155]
2018	DBD (dielectric barrier discharge) plasma	Mouse neuroblastoma Neuro 2A (N2a) cells	activate the Trk/Ras/ERK signaling pathway	Cell Differentiation	[152]
2017	Plasma Jet	SH-SY5Y	Cytoprotection by supplying RONS	Treating diseases in the CNS related to glucose deprivation	[160]
2018	Plasma jet	SH-SY5Y	Neuroprotective effect by NO accumulation	Neuroprotection from hypoxic cell injury	[161]
2019	Plasma Bubbling system	PC12 cells	Neurite growth	Erk and CREB activation	[162]
